# Detailed petrophysical analysis and insights into the Alam El Bueib 3E reservoir from the berenice field, Western desert, Egypt

**DOI:** 10.1038/s41598-025-14708-3

**Published:** 2025-08-12

**Authors:** Amr M. Eid, Walid M. Mabrouk, Mohammed Amer, Ahmed Metwally

**Affiliations:** Geophysics Department, Faculty of Science, Cairo University, Giza, 12613 Egypt

**Keywords:** Geology, Geophysics, Sedimentology, Tectonics

## Abstract

This study presents a comprehensive petrophysical assessment of the Alam El Bueib 3E (AEB-3E) sandstone reservoir in the Berenice Oil Field, located within the Faghur Basin, Western Desert, Egypt. The main objective is to evaluate reservoir quality, hydrocarbon potential, and lateral continuity to support effective field development strategies. The analysis is based on wireline log data from four wells: Berenice-TD-1X, Berenice-03, Berenice-08, and Berenice-09. Lithological analysis using M–N and RHOB–NPHI crossplots confirms that the reservoir is predominantly composed of clean sandstone, with limited shale and siltstone interbeds. Hydrocarbon-bearing intervals were identified between 11,150 and 11,190 feet based on neutron-density separation, resistivity log responses, and indicators of movable hydrocarbons. Formation water resistivity (Rw = 0.0378 Ω·m) and Archie parameters (a = 1, m = 1.9, *n* = 2) were derived from Pickett plot analysis in the Berenice-08 well, providing a basis for water saturation estimation in the absence of core data. The reservoir exhibits low shale content (3–8%), with effective porosity reaching up to 18%, particularly in the southeastern part of the field. Water saturation ranges between 28% and 54%, and the net pay intervals align well with hydrocarbon-bearing zones. Structural mapping and well correlation indicate consistent reservoir thickness, with central thickening influenced by ENE–WSW trending normal faults. Seismic interpretation reveals horst and graben structures that contribute to reservoir compartmentalization. The petroleum system is supported by mature source rocks of the Safa Formation, in addition to effective intraformational and regional seals, which enhance hydrocarbon entrapment. The results of this study contribute to a clearer understanding of the petrophysical and structural characteristics of the AEB-3E reservoir, offering valuable insights for future development and exploration efforts in the region.

*Key-words: Petrophysical evaluation*,* AEB-3E reservoir*,* Berenice Oil Field*,* hydrocarbon detection*,* structural trapping*.

## Introduction

The Western Desert of Egypt represents one of the most significant hydrocarbon provinces in North Africa. Geologically, it is characterized by a complex tectonic evolution influenced by multiple phases of extension and compression. The region is subdivided into several basins and uplifts, including the Faghur, Matruh, Shushan, and Abu Gharadig basins, which have been shaped primarily by Mesozoic and Cenozoic tectonics. These basins exhibit well-developed rift systems and structural traps that have made the Western Desert a key target for oil and gas exploration^1–3^. The combination of favorable source rocks, reservoir units, and effective seals has led to prolific petroleum systems and continuous hydrocarbon discoveries.

**Fig. 1:**
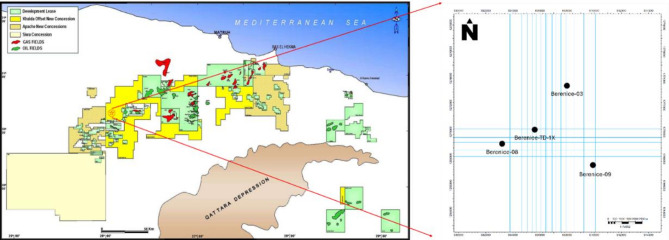
Base map of the Berenice Oil Field illustrating the study area and the locations of the four analyzed wells^18^.

Among the productive regions within the Western Desert is the Berenice Field, located in the northeastern part of the Faghur Basin (Figure. 1). This field has gained attention due to its sustained hydrocarbon output, structural complexity, and well-established infrastructure. Its strategic location within a mature petroleum basin, coupled with high-quality reservoir rocks, positions it as a critical component of Egypt’s energy development plans^4^. Continuous drilling and exploration efforts have revealed promising reservoir intervals, particularly within the Lower Cretaceous sequences, which have shown excellent reservoir potential and significant recoverable reserves.

The Alam El Bueib Sub-member, of Lower Cretaceous age, is one of the principal reservoirs in the Western Desert, consisting mainly of alternating sandstone, siltstone, shale, and carbonate units. It is subdivided into several members, notably AEB-6, AEB-5, AEB-4, AEB-3 (including sub-members such as AEB-3 A, AEB-3B, and AEB-3E), and AEB-2. These members were deposited in fluvio-deltaic to shallow marine environments, leading to significant heterogeneity in lithofacies and reservoir quality. The AEB-3E unit, in particular, has shown great promise as a productive sandstone interval, and its petrophysical behavior plays a crucial role in determining the field’s productivity^5–7^.

Several studies have addressed the stratigraphy, depositional settings, and reservoir characteristics of the Alam El Bueib (AEB) Sub-member across the North Western Desert of Egypt, highlighting its significance as a key Lower Cretaceous reservoir. The AEB Sub-member is widely recognized for its stacked sandstone sequences, deposited in fluvio-deltaic to shallow marine environments, with varying degrees of heterogeneity and reservoir quality^8,9^. In the Shushan and Matruh basins, detailed sedimentological and petrophysical studies have been conducted to assess reservoir potential and compartmentalization^10,11^. In the vicinity of the Faghur Basin, limited published work exists, but some studies have documented the lateral facies changes, fault-controlled thickness variations, and potential hydrocarbon accumulations within the AEB intervals^12–14^. However, the Berenice Field remains underrepresented in the literature, and detailed petrophysical evaluations integrating structural interpretation are scarce. This study aims to address this gap by providing a comprehensive analysis of the AEB-3E reservoir in the Berenice Oil Field, contributing to a better understanding of its hydrocarbon potential and regional development.

Petrophysical analysis is a cornerstone in reservoir evaluation, providing critical insights into rock properties such as porosity, permeability, water saturation, and lithology. Understanding these parameters is essential for determining hydrocarbon-bearing zones, estimating reserves, and optimizing production strategies. Integrated analysis using well logs, core data, and geological models allows for a more precise characterization of reservoir behavior and heterogeneity, which is especially important in complex formations like the AEB-3E. Furthermore, correlating petrophysical data across wells contributes to a comprehensive understanding of lateral and vertical facies variations^11,15–17^.

This study aims to conduct a detailed petrophysical evaluation of the Alam El Bueib 3E reservoir in the Berenice Field. The primary objectives are to characterize the reservoir matrix, assess the key petrophysical parameters (such as porosity, permeability, and saturation), and evaluate the hydrocarbon potential of this unit. The results are mapped to visualize the spatial distribution of these parameters across the study area, providing valuable insights into reservoir continuity and quality. In addition, well-to-well correlations are used to confirm the lateral extent and stratigraphic consistency of the AEB-3E sub-member, thereby supporting effective field development and management.

## Geological settings

**Fig. 2:**
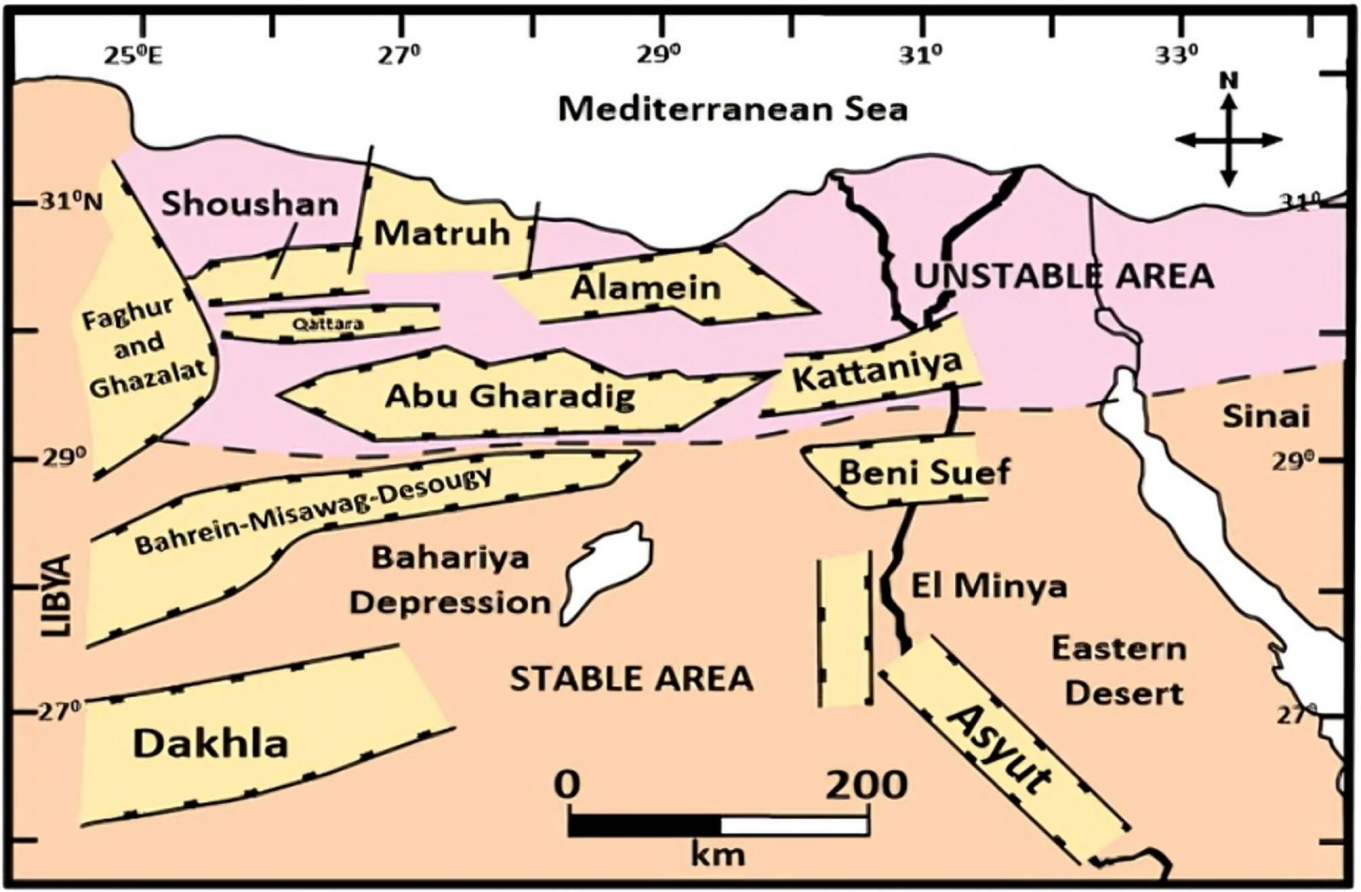
Simplified geological map of the Northern Western Desert of Egypt, highlighting major structural elements and sedimentary basins, including the location of the Faghur Basin where the Berenice Field is situated.

The Faghur Basin, situated in the southern part of the Matruh Basin in Egypt’s Western Desert (Figure. 2), has undergone a complex tectonic history influenced by multiple extensional and compressional phases. This intra-cratonic basin developed primarily due to rifting events associated with the early Mesozoic break-up of Gondwana. The dominant structural elements include NE–SW and WNW–ESE trending normal faults, which were later overprinted by transpressional features during Late Cretaceous and Paleogene tectonic events. These structural regimes have shaped the basin’s compartmentalization and created numerous fault-bounded traps that control hydrocarbon accumulation and migration pathways^1–3,18,19^.

The Berenice Field, located in the northeastern Faghur Basin, lies within an area that experienced significant tectonic uplift and subsidence episodes. The field is structurally complex, with tilted fault blocks formed during the Jurassic to Early Cretaceous rifting, followed by inversion tectonics during the Syrian Arc compressional phase in the Late Cretaceous to Paleogene. The prevailing fault trends in the field are predominantly NW–SE and NE–SW, reflecting the underlying rift architecture. These faults have a direct impact on reservoir continuity, fluid distribution, and trap integrity, making structural analysis vital to any exploration and development activities in the area^4,18^.

**Fig. 3:**
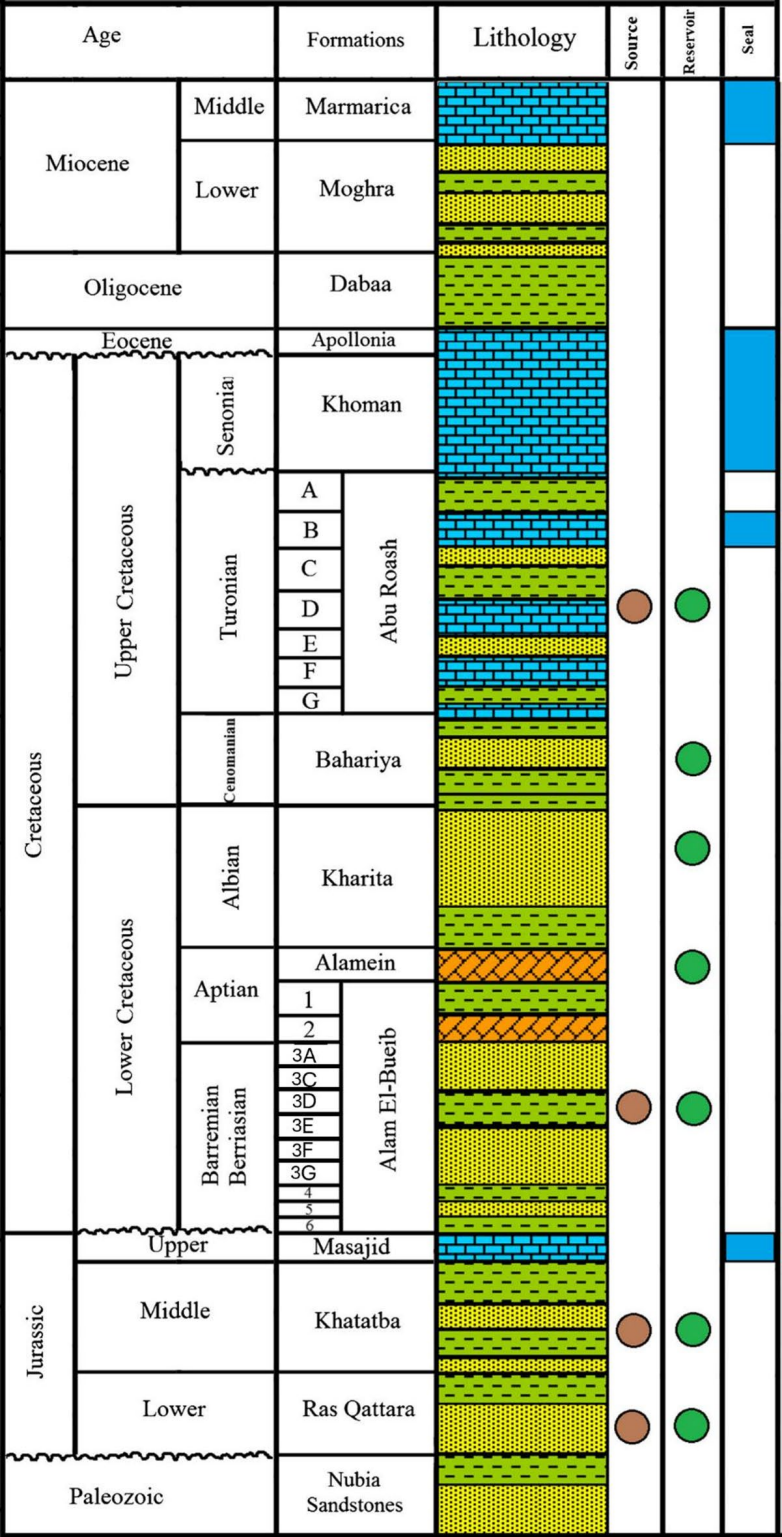
Stratigraphic column representing the geological succession in the northern sector of Egypt’s Western Desert^29^.

Stratigraphically, the area exhibits a complete sedimentary succession ranging from the Precambrian to the Cenozoic^25^. The Precambrian basement, consisting of igneous and metamorphic rocks, forms the structural foundation of the basin. Above it lies the Paleozoic sequence, which is relatively thin and poorly developed in this region^20–23^. The Mesozoic succession begins with the Jurassic Khatatba Formation, composed mainly of interbedded shales and sandstones, and is recognized as a major source rock due to its rich organic content. Overlying it is the Lower Cretaceous Alam El Bueib Formation, consisting of sandstones, siltstones, and shales, and acting as a key reservoir unit in many Western Desert fields, including Berenice (Figure. 3).

The Bahariya Formation follows, made up of fluvial to shallow marine sandstones and shales, and is also an important hydrocarbon reservoir. Above it lies the Abu Roash Formation of Upper Cretaceous age, which includes several members with alternating carbonate and shale lithologies. The Abu Roash “F” Member is a notable source rock, while the “G” Member often acts as an effective seal. The overlying Khoman Formation, composed primarily of chalky limestones, represents a regional seal rock due to its low permeability. The Paleocene to Eocene succession includes the Dabaa, Apollonia, and other formations that are mainly carbonates and marls, providing additional sealing capacity and overburden pressure for underlying reservoirs^1,2,24^.

The Alam El Bueib Sub-member, of Early Cretaceous age, is subdivided into several reservoir-bearing members including AEB-6, AEB-5, AEB-4, AEB-3 (which includes sub-members AEB-3 A, AEB-3B, and AEB-3E), and AEB-2^25,26^. These members represent varied depositional settings ranging from fluvial-deltaic to shallow marine environments. The AEB-3 unit is particularly important in many fields of the Western Desert due to its consistent reservoir quality and favorable thickness. Within this unit, the AEB-3E sub-member stands out as a significant hydrocarbon-bearing interval, characterized by alternating sandstone and shale layers. Its depositional environment reflects a transitional marine influence, which has contributed to good reservoir development in terms of both porosity and permeability. The heterogeneity between the sandstone bodies and interbedded shales poses both opportunities and challenges for reservoir exploitation and highlights the need for detailed petrophysical analysis.

The Alam El Bueib 3E (AEB-3E) unit, within the Alam El Bueib Formation, is the main focus of this study. It comprises fine- to medium-grained sandstones interbedded with shales and occasional siltstones, deposited in a shallow marine to deltaic environment. This interval exhibits significant petrophysical variability due to facies changes, diagenetic alterations, and the influence of structural heterogeneity^12,26–28^. The AEB-3E reservoir has proven to be one of the most productive zones in the Berenice Field, with favorable porosity and permeability in several intervals. Understanding the distribution, matrix properties, and reservoir behavior of this unit is essential for optimizing hydrocarbon recovery and guiding future development strategies in the field.

## Data and methodology

This study utilizes comprehensive wireline log data acquired from four key wells within the Berenice Field, located in the Faghur Basin, Western Desert, Egypt. The wells included in the analysis are Berenice-TD-1X, Berenice-03, Berenice-08, and Berenice-09. The dataset encompasses a suite of conventional wireline logs including Gamma Ray (GR), Density (RHOB), Neutron Porosity (NPHI), Sonic (∆t), and multiple resistivity measurements (MSFL, LLS, LLD). These logs serve as the foundation for the petrophysical evaluation of the Alam El Bueib 3E reservoir, enabling detailed lithological discrimination and assessment of reservoir quality and hydrocarbon potential.

**Fig. 4:**
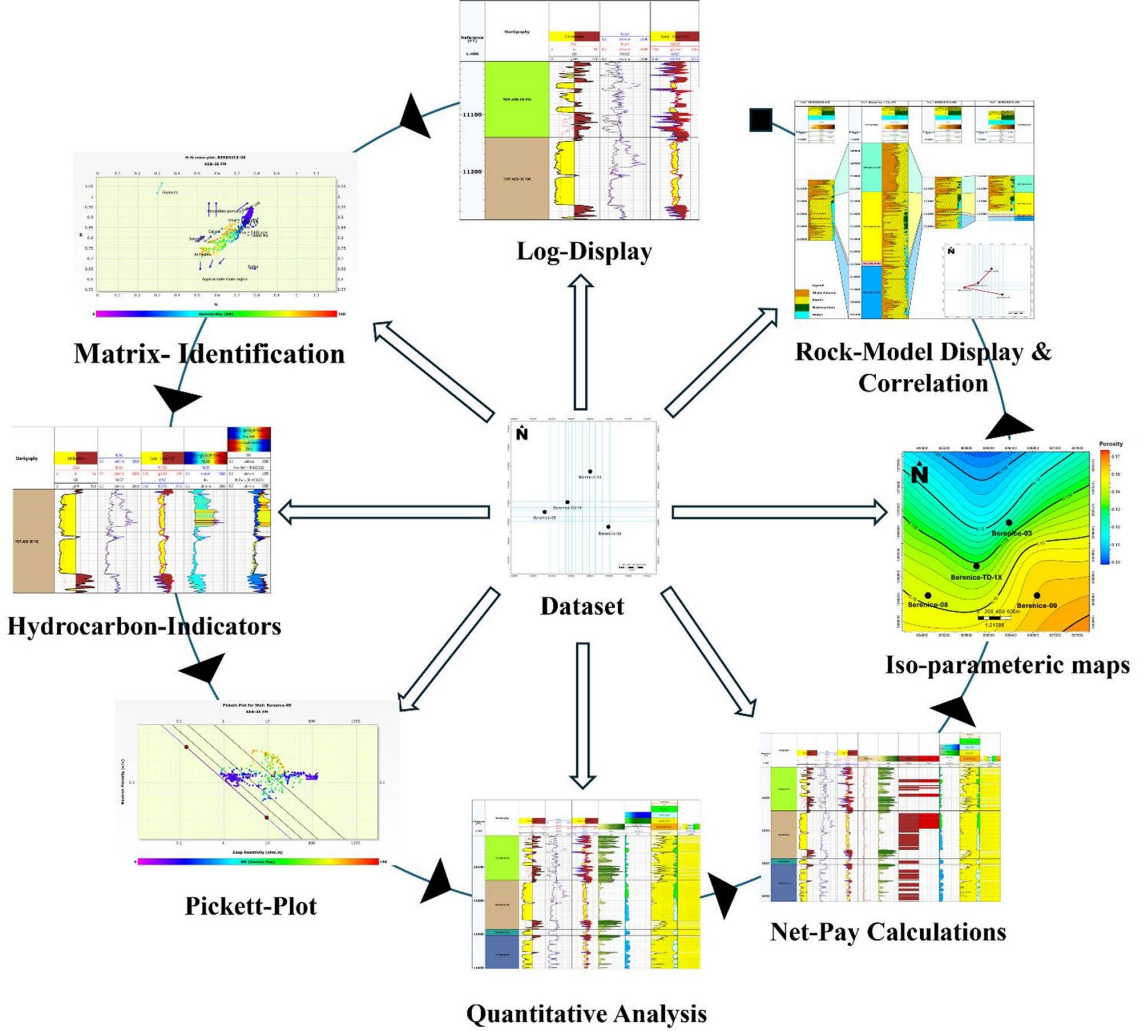
Workflow of the petrophysical analysis, showing steps from data preparation to log display, matrix and hydrocarbon identification, parameter calculation, mapping, and well correlation for the AEB-3E reservoir in Berenice Field.

The methodology adopted in this study follows a structured workflow (Fig. 4) that begins with data preparation, quality control, and log visualization, followed by both qualitative and quantitative petrophysical analyses. This integrated workflow ensures a robust interpretation of subsurface properties and aids in building a reliable reservoir model. The key steps of the petrophysical analysis are designed to characterize the matrix composition, identify hydrocarbon-bearing zones, and evaluate the distribution of petrophysical parameters essential for reservoir evaluation.

The first step in the workflow involves Log Data Display, where log data from the selected wells are cleaned, conditioned, and visually inspected. This step is critical for understanding the basic lithological patterns and identifying potential reservoir and non-reservoir intervals. Following this, Matrix Identification is performed using cross-plot techniques and integrated log interpretation, particularly employing the density, neutron, and photoelectric logs to determine the dominant lithologies and mineralogical composition across the reservoir interval.

Next, the study proceeds with Hydrocarbon Identification, utilizing key log indicators such as high resistivity responses, separation between neutron and density logs, and characteristic GR signatures to delineate hydrocarbon-bearing zones. This is followed by constructing the Pickett Plot, which is used to determine the formation water resistivity (Rw) and validate hydrocarbon saturation estimates. Subsequently, quantitative calculations are performed for Volume of Shale (Vsh), Effective Porosity (ϕe), and Water Saturation (Sw), forming the core of the petrophysical evaluation.

To define the net-pay intervals, specific cut-off values were applied based on regional analogs and reservoir characteristics. The following thresholds were used: porosity ≥ 10%, water saturation ≤ 50%, and shale volume ≤ 30%. These values were selected to distinguish zones with sufficient reservoir quality and hydrocarbon saturation that are considered potentially productive. These cut-offs were consistently applied across all wells to ensure uniformity in identifying net pay and were supported by published studies on analogous reservoirs in the Western Desert.

In the final stages, a Rock Model Display is constructed to visualize and integrate key petrophysical outputs. This model displays vertical profiles of volume of shale, effective porosity, bulk volume of water (BVW), and bulk volume of hydrocarbon (BVH). These calculated parameters are then used to generate areal maps for formation thickness, volume of shale, effective porosity, and water saturation, highlighting the spatial distribution of reservoir quality across the study area. Lastly, Well Correlation is carried out between the four wells to delineate the lateral extent and continuity of the Alam El Bueib 3E reservoir, providing insights into reservoir heterogeneity and aiding in the prediction of prospective zones.

## Results and discussion

### Log display & key zones

The initial phase of the petrophysical analysis involved the thorough review and quality control of the available well log data from the Berenice Field^11,30–32^. This step was essential to ensure the reliability and consistency of the measurements across the selected wells (Berenice-TD-1X, Berenice-03, Berenice-08, and Berenice-09). The log display process included a detailed examination of the main wireline logs: Gamma Ray (GR), Density (RHOB), Neutron Porosity (NPHI), Sonic (∆T), and Resistivity logs to identify any anomalies, gaps, or inconsistencies that might affect the interpretation. Verifying the integrity of the log responses before proceeding with the analysis provided a solid base for deriving accurate petrophysical parameters. Figure 4 shows the composite log display for the Berenice-08 well, which served as a reference for cross-checking the quality and consistency of data across the study area.

**Fig. 5:**
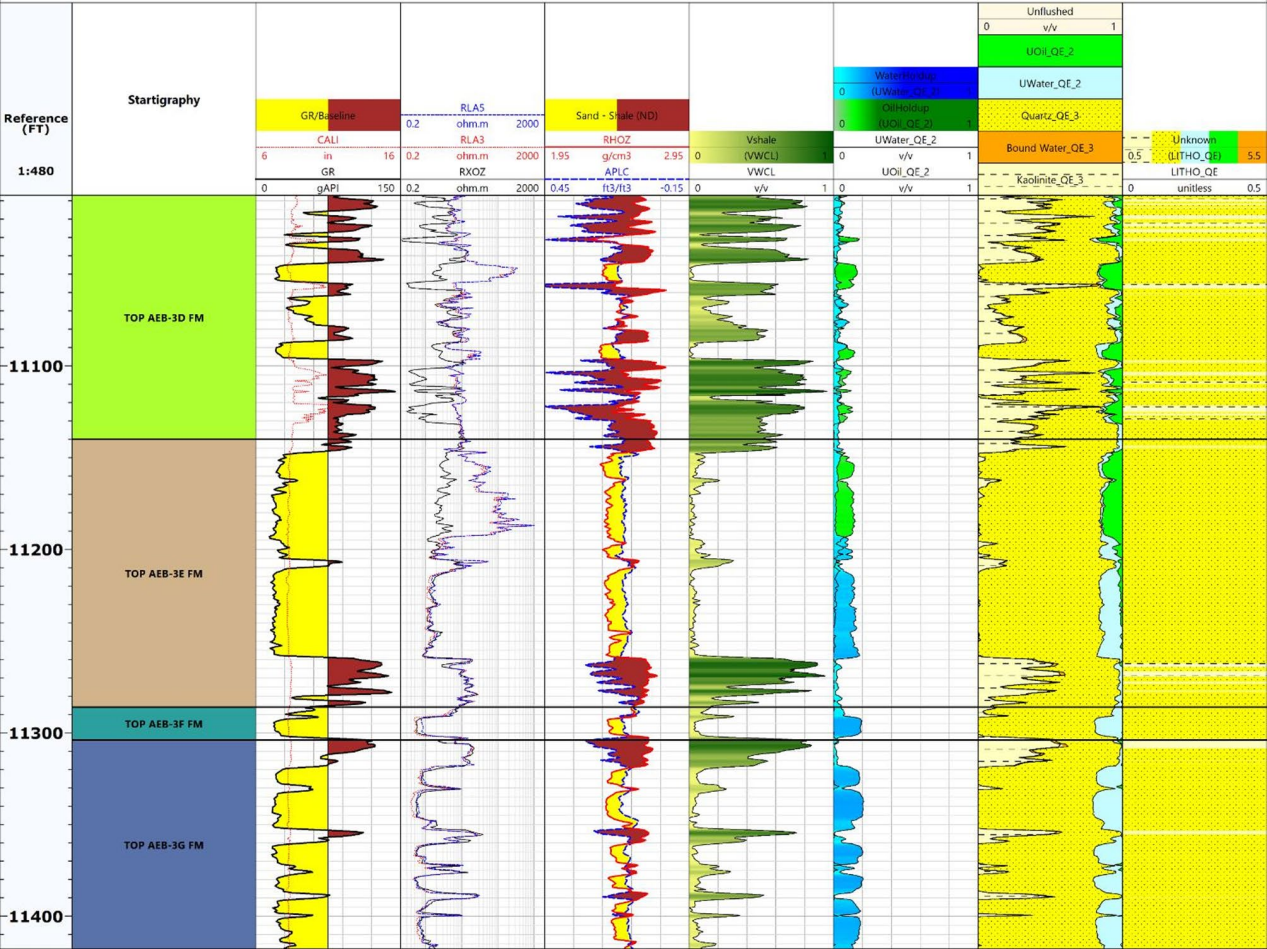
Composite presentation of primary wireline logs for Well Berenice-08, including Gamma Ray, Density, Neutron Porosity, Sonic, and Resistivity measurements.

The identification of the primary reservoir zones in Berenice Field was carried out by analyzing the log responses across the studied wells. This process enabled the recognition of distinct lithological units and their associated reservoir characteristics. Special focus was directed toward the Alam El Bueib 3E Member due to its notable petrophysical properties, including relatively high porosity and resistivity values, which indicate promising hydrocarbon potential. These attributes mark the 3E Member as a key target for further evaluation and development. Other intervals within the Alam El Bueib Sub-member were also examined to understand their lithological variability and fluid distribution, which play a critical role in hydrocarbon entrapment. Through this analysis, the most prospective zones were identified for detailed petrophysical evaluation. Refer to Fig. 5.

### Matrix identification

The lithology and matrix composition of the Alam El Bueib 3E reservoir in the Berenice Field were determined using two distinct petrophysical crossplot techniques: the M-N crossplot and the RHOB-NPHI crossplot. These methods complement each other and enhance the reliability of mineralogical interpretation.

**Fig. 6:**
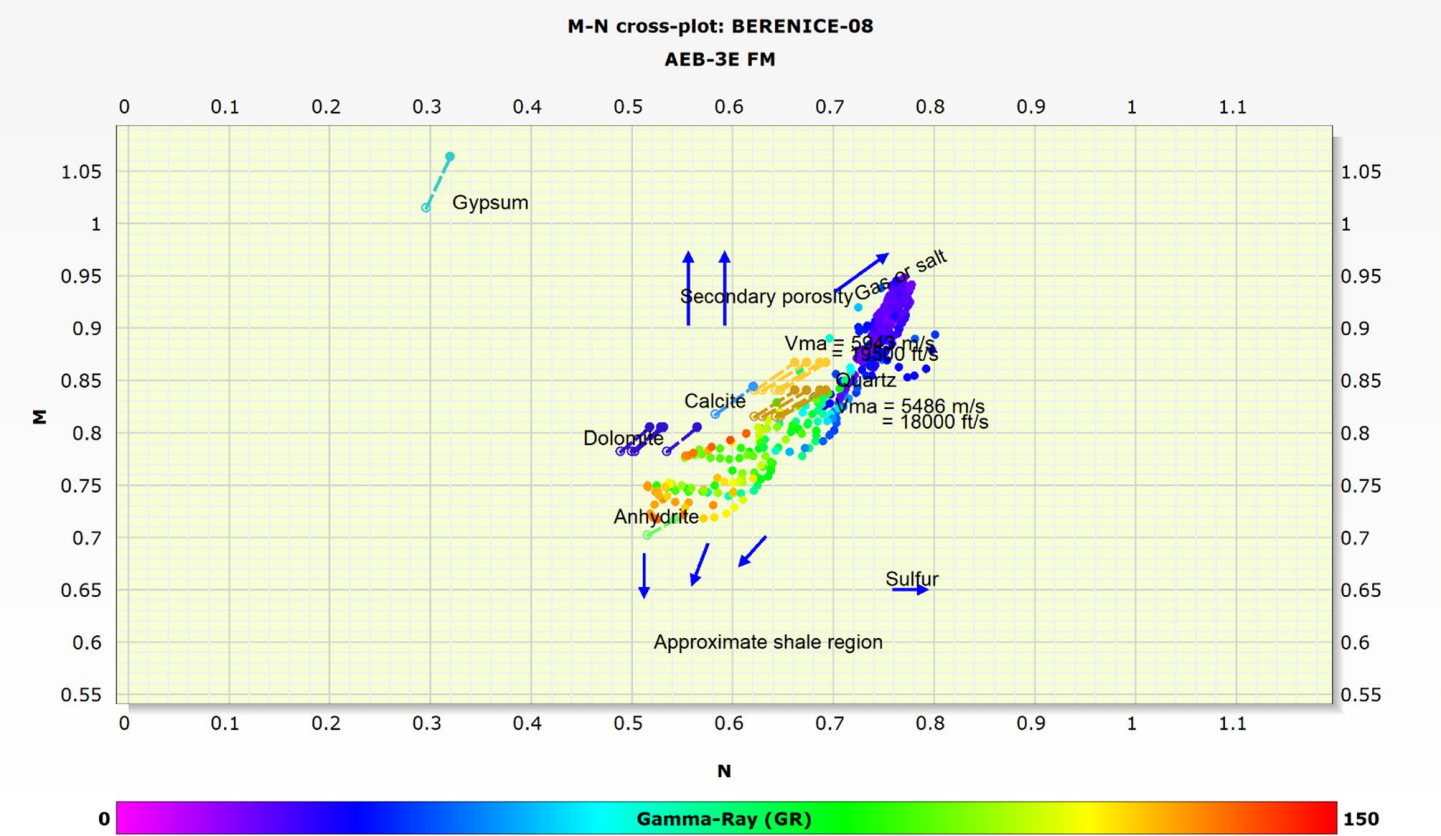
M–N crossplot for the Alam El Bueib 3E Member, illustrating matrix composition and lithological characteristics.

The M-N crossplot integrates sonic (ΔT), density (RHOB), and neutron porosity (NPHI) log responses to derive M and N values. When plotted, these values allow for the differentiation between key lithologies such as quartz, calcite, dolomite, and clay. The data from the wells studied consistently along with the expected lithological trends, providing a clear understanding of the formation’s mineral composition. This method was particularly useful in detecting mixed lithologies and subtle mineral variations^11,16,17,33^. Figure 6 presents the M-N crossplot results for the Alam El Bueib 3E Member. Based on the M-N crossplot analysis, the AEB-3E Sub-member is primarily composed of sandstone, with additional presence of siltstone and shale, as indicated by the clustering of data points along the respective lithology trends. The sandstone points also show a notable shift toward the gas direction, reflecting hydrocarbon-bearing characteristics within the formation.

**Fig. 7:**
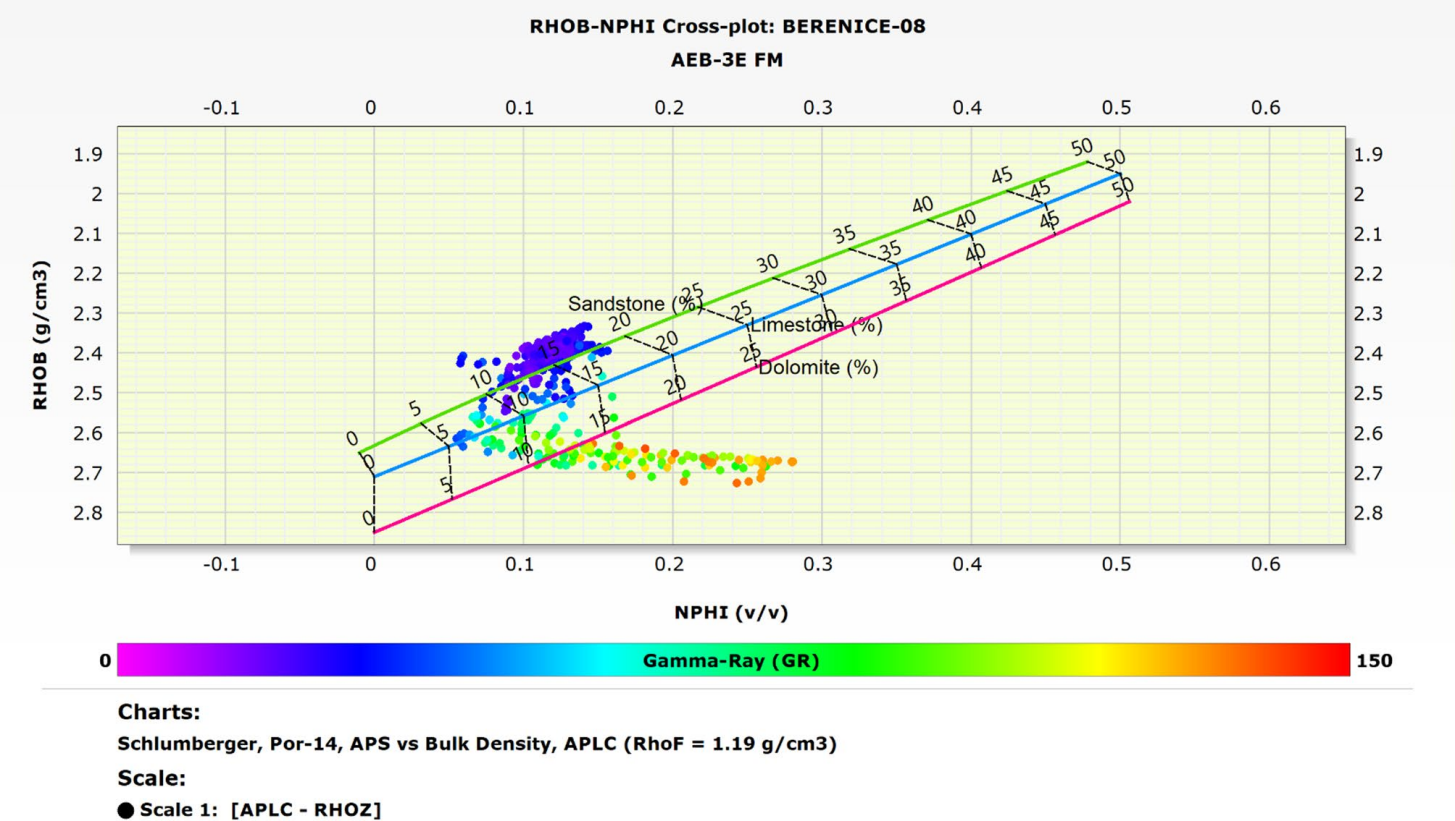
RHOB–NPHI crossplot for the Alam El Bueib 3E Member, illustrating porosity trends and lithological variations.

In parallel, the RHOB-NPHI crossplot was utilized to validate and support the findings from the M-N crossplot. This traditional and straightforward method plots bulk density against neutron porosity, producing lithology trends that are easy to interpret. Distinct clusters on this plot correspond to specific rock types, enabling a quick assessment of clean sands, carbonates, and shaly intervals. The RHOB-NPHI crossplot helped confirm the presence of the identified lithologies and refine the matrix classification^15,28,30,34^. Figure 7 displays the RHOB-NPHI crossplot for the studied interval. Interpreting the RHOB-NPHI crossplot further confirms this composition, where there is clear clustering of data points along the sandstone reference line, with additional dispersion toward the shale and siltstone regions. This pattern indicates that the formation is primarily composed of clean sandstone, with interbedded siltstone and shale layers.

By integrating both crossplot techniques, a comprehensive and consistent matrix identification was achieved, which laid the foundation for more accurate porosity, saturation, and reservoir quality evaluations in subsequent analysis steps.

### Hydrocarbon indicators

Hydrocarbon detection within the Alam El Bueib 3E reservoir in the Berenice Field was conducted using three integrated methods: (1) neutron-density crossover, (2) resistivity separation (Rt–Ro), and (3) the movable oil plot^35–37^. The neutron-density crossover technique involves comparing neutron porosity (NPHI) and bulk density (RHOB) logs. A noticeable crossover, where the neutron reading exceeds the density, indicates the presence of hydrocarbons, gas typically shows a larger separation, while oil exhibits a smaller one. This helped delineate hydrocarbon-bearing intervals across the studied wells.

The second method, resistivity separation, involves comparing true formation resistivity (Rt) and flushed zone resistivity (Ro). A higher Rt than Ro suggests the presence of hydrocarbons, as hydrocarbon-bearing zones tend to have higher electrical resistivity due to low water saturation. The third approach utilized was the movable oil plot, which integrates key resistivity-derived ratios: the formation resistivity factor (FRF = Rt/Φ²), Rxo/Rmf, and Rt/Rw. The gap between FRF and Rxo/Rmf is interpreted as residual hydrocarbons, while the gap between Rxo/Rmf and Rt/Rw indicates movable hydrocarbons. This method enhances the understanding of hydrocarbon mobility and saturation within the formation.

**Fig. 8:**
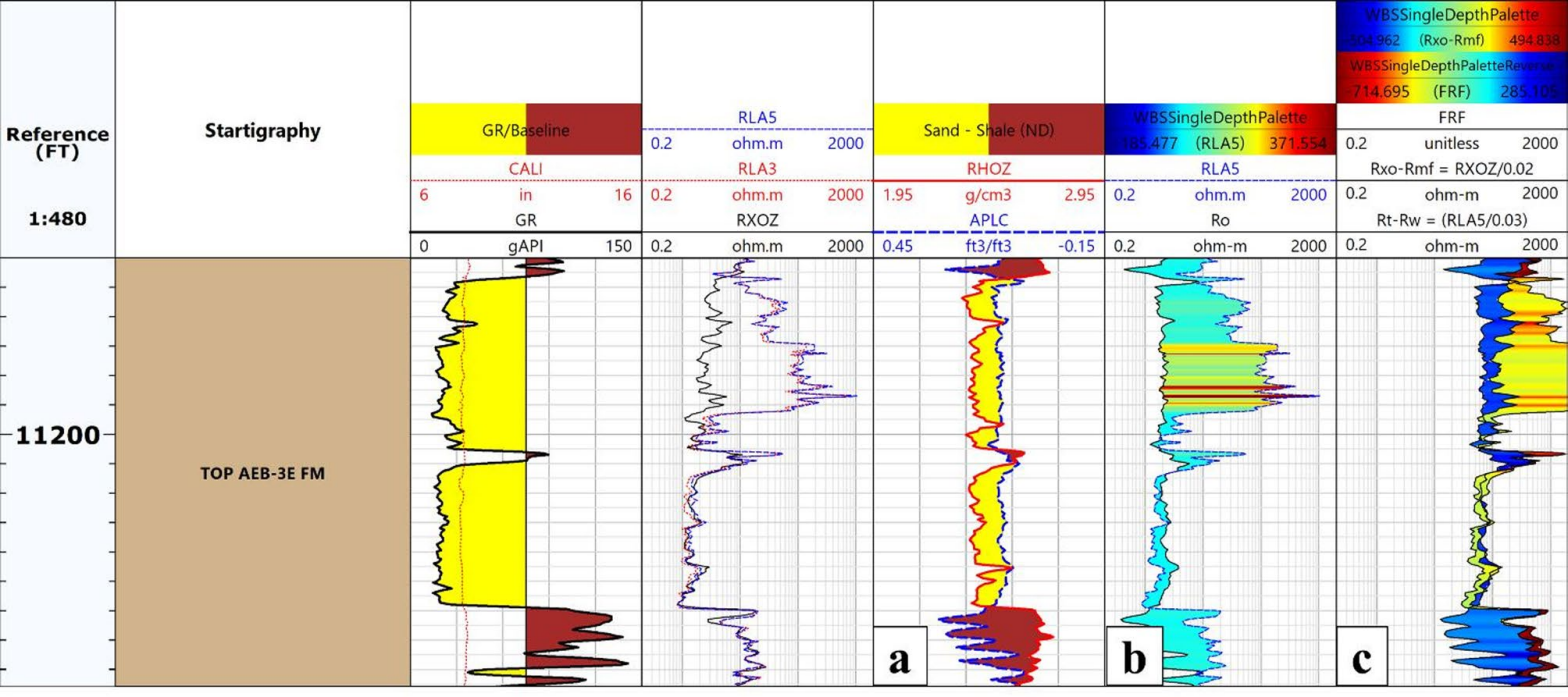
Hydrocarbon detection in the Alam El Bueib 3E Member of the Berenice-08 well using integrated well log analysis: (**a**) Neutron–Density crossover, (**b**) True vs. flushed zone resistivity (Rt–Ro) overlay, and (**c**) Movable oil plot.

By integrating the three applied techniques, the presence of hydrocarbons within the depth interval of 11,150 to 11,190 ft has been confidently confirmed. Figure 8 demonstrates the implementation of these methods in the Berenice-08 well, providing valuable insights that support accurate reservoir evaluation and effective development planning.

### Pickett plot analysis

In the absence of core-derived parameters, the Pickett plot serves as a crucial method for estimating key electrical properties of the reservoir^38,39^. For the Berenice Field, this analysis was applied to the Berenice-08 well to determine the formation water resistivity (Rw), along with the tortuosity factor (a), cementation exponent (m), and saturation exponent (n), essential inputs for accurate water saturation calculations.

The Pickett plot involves plotting deep resistivity (Rt) against porosity (Φ) on a logarithmic scale. This relationship helps visually identify fluid types in the formation and derive Rw by extrapolating the trend line through 100% water-saturated zones. Additionally, the slopes and intercepts of the plotted data allow for the estimation of the Archie parameters (a, m, and n), which are necessary for characterizing the electrical behavior of the rock matrix.

**Fig. 9:**
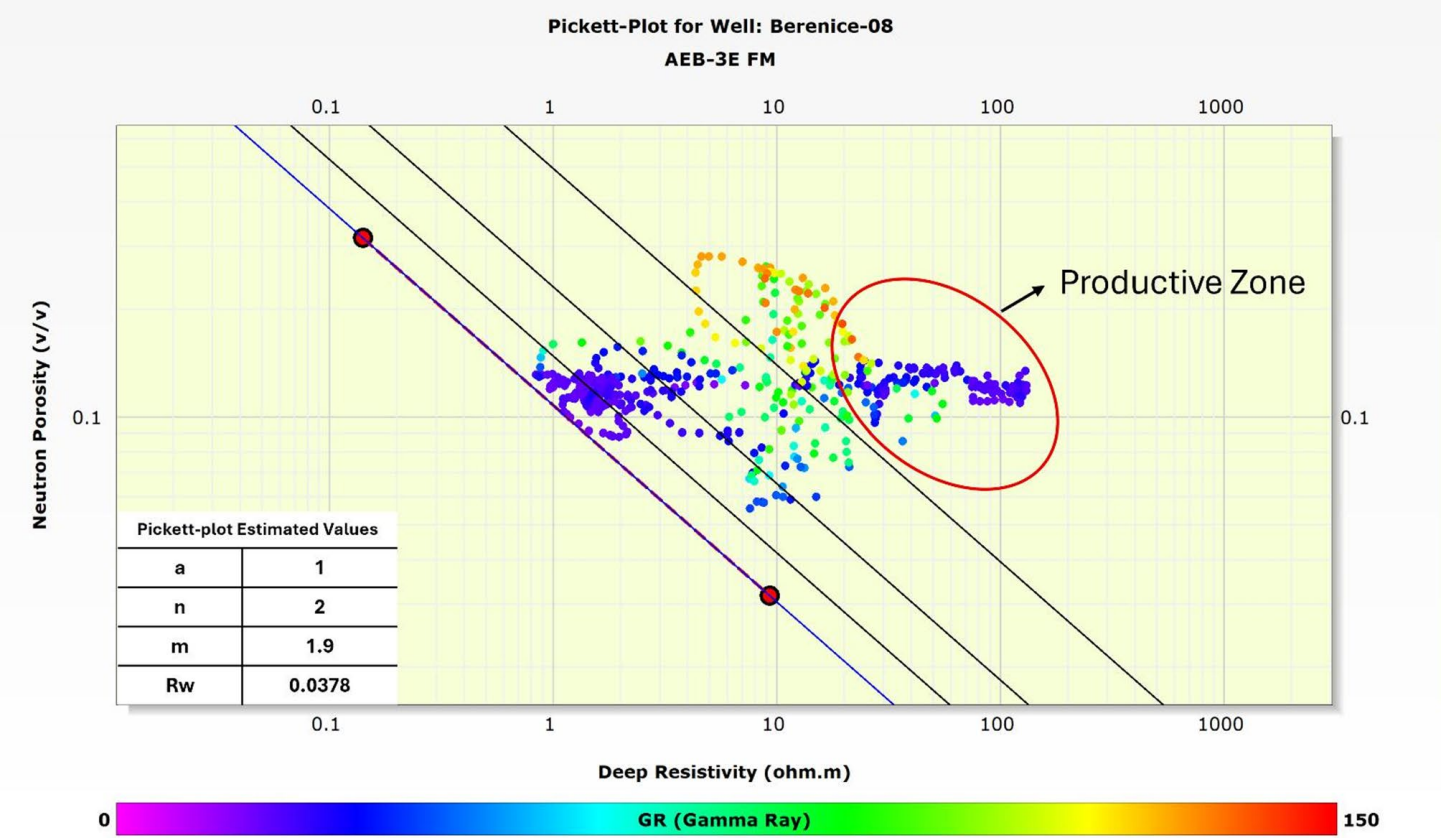
Pickett plot for the Berenice-08 well, with red circle highlighted the productive zone, illustrating the derived reservoir parameters, including formation water resistivity and Archie constants.

By utilizing this method, we were able to overcome the lack of core data and obtain reliable estimations of reservoir electrical properties, which directly influence hydrocarbon saturation calculations and reservoir quality assessment. Figure 9 illustrates the Pickett plot constructed for the Berenice-08 well showing the values of each parameter calculated.

### Petrophysical parameters calculation

**Fig. 10:**
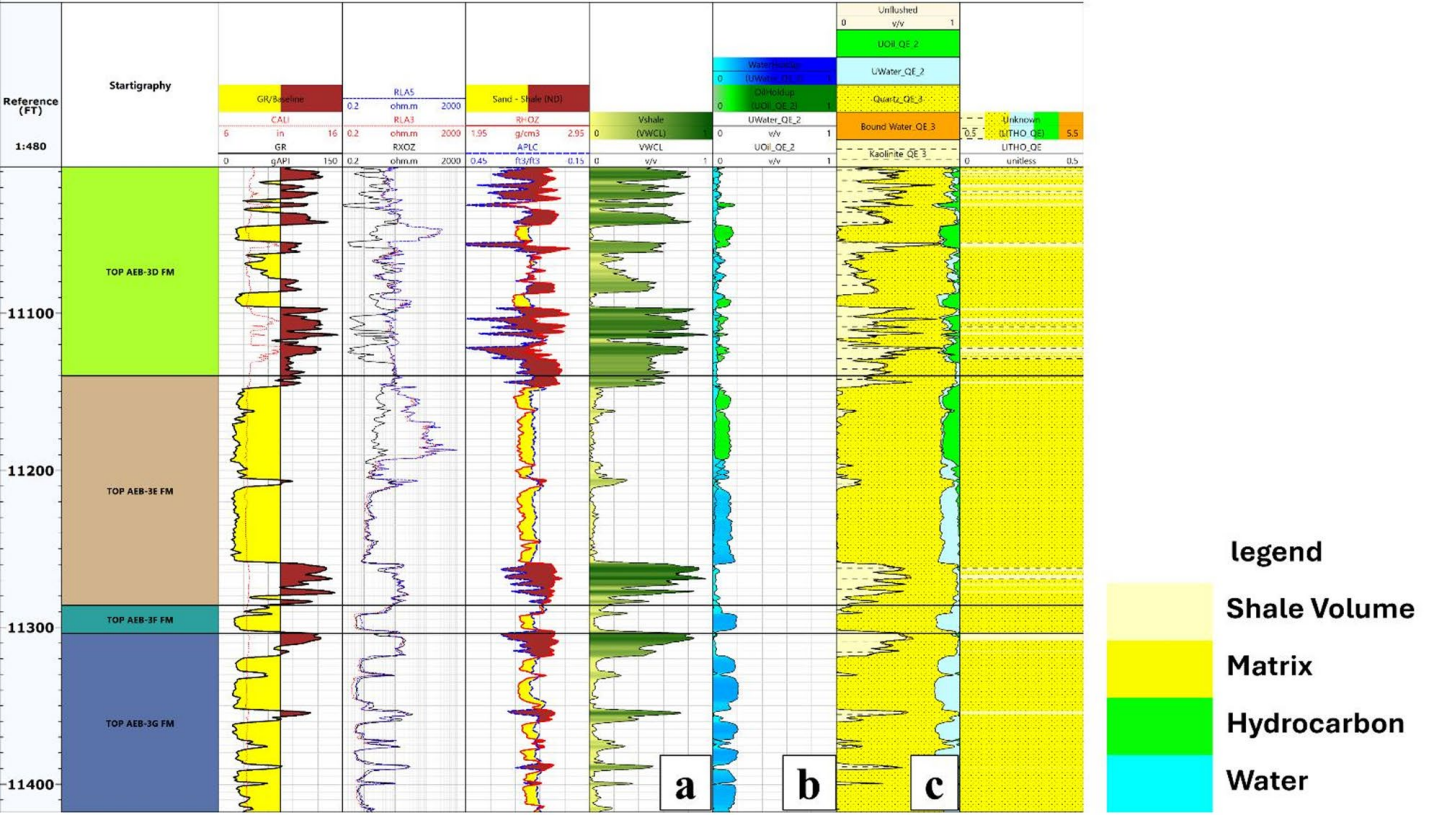
Petrophysical analysis results for the Berenice-08 well, presenting (**a**) calculated shale volume, (**b**) water and hydrocarbon saturations, and (**c**) an integrated rock model displaying shale volume, bulk volumes of water and hydrocarbons, and matrix volume.

A systematic workflow was implemented to evaluate the petrophysical characteristics of the Berenice Oil Field, with a focus on the AEB-3E sub-member (Figure. 10). This workflow involved a step-by-step calculation of essential reservoir properties—namely shale volume, total and effective porosity, water saturation, bulk volume of water, and bulk volume of hydrocarbons. Each parameter plays a critical role in assessing reservoir quality and hydrocarbon potential.

#### Volume of shale (Vsh)

The initial step involved estimating the Volume of Shale (Vsh), which is crucial for distinguishing between clean and shaly formations. Vsh was derived from Gamma Ray (GR) logs using standard empirical relationships. To improve accuracy, especially in intervals with dispersed clay, the calculated gamma ray index was corrected using the Stieber equation, which accounts for the non-linear relationship between GR response and shale content in shaly sand reservoirs. This correction provides a more realistic estimation of Vsh, particularly in formations where shale is finely distributed rather than laminated. This parameter is essential because excessive shale content often correlates with poor reservoir quality due to its impact on porosity, permeability, and fluid mobility. Quantifying Vsh helps correct subsequent petrophysical parameters by isolating the non-reservoir clay fraction within the rock. The Volume of Shale was calculated using the following formulas^40^:

1 $$\:{I}_{GR}\:=\frac{{GR}_{log}-\:{GR}_{min}}{{GR}_{max}\:-\:{GR}_{min}}$$

2 $$\:Vsh\:=\frac{{I}_{GR}}{3\:-\:2{I}_{GR}}\:$$

Where:$\:{I}_{GR}$: Gamma Ray index, $\:{GR}_{log}$​: measured Gamma Ray value from the log, $\:{GR}_{min}$​: minimum GR value representing clean formation, $\:{GR}_{max}$​: maximum GR value indicating shale and $\:Vsh$: Corrected volume of shale with Steiber equation.

#### Total and effective porosity (ΦT and Φeff)

Following shale volume estimation, the Total Porosity (ΦT) was calculated by integrating Density (RHOB) and Neutron (NPHI) log data. The total porosity represents the entire pore volume within the rock, regardless of its ability to transmit fluids. However, not all porosity contributes to hydrocarbon flow, especially in shaly zones.

To account for this, Effective Porosity (Φeff) was then determined by excluding the porosity associated with shale content. This correction provides a more accurate measure of the pore space available for movable hydrocarbons, which directly influences reservoir performance and production potential. The calculation began by estimating Total Porosity (ΦT​) using both the Density (RHOB) and Neutron (NPHI) logs. Following the method outlined by Asquith and Gibson (1982), total porosity was derived from the average of the porosity values obtained from these two logs, the Density Porosity (ΦD​) was calculated and Effective Porosity (Φeff), the effect of shale content was removed from the total porosity. Equations of these three logs are illustrated below^40,41^.

3 $$\:{{\varnothing}}_{T}=\:\frac{{{\varnothing}}_{N}+\:{{\varnothing}}_{D}\:}{2}$$

4 $$\:{{\varnothing}}_{D}=\frac{{\rho\:}_{m}-{\rho\:}_{b}}{{\rho\:}_{m}-\:{\rho\:}_{fl}}\:$$

5 $$\:{{\varnothing}}_{eff}=\:{{\varnothing}}_{T}-\left(\:{V}_{sh}\text{*}\:{{\varnothing}}_{sh}\right)$$

where ρma is the matrix density, $\:{\rho\:}_{b}$ is the bulk density obtained from the log, and $\:{\rho\:}_{fl}$ represents the fluid density, usually that of formation water. Additionally, $\:{{\varnothing}}_{eff}$ denotes effective porosity, $\:{{\varnothing}}_{T}$ is the total porosity, $\:{V}_{sh}$ indicates the shale volume, and $\:{{\varnothing}}_{sh}$ corresponds to the porosity of the shale fraction.

#### Water saturation (Sw)

The final step involved computing Water Saturation (Sw), which indicates the fraction of the pore space occupied by formation water. Sw was estimated using resistivity logs in combination with the Archie equation, with inputs such as porosity, formation water resistivity (Rw), and true formation resistivity (Rt). Accurate Sw estimation is vital for differentiating hydrocarbon-bearing zones from water-saturated intervals and for calculating hydrocarbon saturation (Sh), which is inversely related to Sw.

6 $$\:\frac{1}{\sqrt{{R}_{t}}}=\left[\sqrt{\frac{{\phi\:}^{m}}{a{R}_{w}}}+\frac{{{V}_{cl}}^{\left(\frac{1-{V}_{cl}}{2}\right)}}{\sqrt{{R}_{cl}}}\right]{{S}_{w}}^{n}$$

Water saturation (Sw) can be calculated using the previous Eq. 4^7^. In this formula, $\:{S}_{w}$ represents water saturation, $\:{R}_{t}$ is the true resistivity, and $\:{V}_{cl}$ indicates the volume of clay or shale. Additionally, $\:{{\varnothing}}_{T}$ denotes total porosity, $\:{{\varnothing}}_{sh}$ is the porosity of the shale, *a* is the tortuosity factor, $\:{R}_{w}$ corresponds to the resistivity of formation water, and $\:{R}_{cl}$ represents the resistivity of the shale.

#### Bulk volume of water (BVW) and bulk volume of hydrocarbons (BVH)

Beyond saturation, Bulk Volume of Water (BVW) and Bulk Volume of Hydrocarbon (BVH) were calculated to quantify the actual volume of fluids present within the reservoir rock. BVW is the product of effective porosity and water saturation, representing the volume of pore space filled with water. Conversely, BVH represents the pore volume occupied by hydrocarbons and is derived by multiplying effective porosity by hydrocarbon saturation (Sh). These volumes provide a direct measure of the fluid distribution within the reservoir, essential for estimating reserves and planning production. This can be calculated using the equation provided below^42^:

7 $$\:BVW=SW\text{*}\:{{\varnothing}}_{eff}$$

8 $$\:BVHC=SHC\text{*}\:{{\varnothing}}_{eff}$$

In this equation, $\:BVW$ stands for bulk volume of water, Sw represents water saturation, $\:{{\varnothing}}_{eff}$ refers to effective porosity, $\:BVHC$ denotes bulk volume of hydrocarbons, and $\:SHC$ indicates hydrocarbon saturation.

### Petrophysical analysis

A comprehensive petrophysical evaluation (Table 1) was carried out across all wells in the Berenice Oil Field, with a particular focus on the AEB-3E sub-member. The interpretation process utilized wireline log data, including Gamma Ray (GR), Neutron (NPHI), Density (RHOB), and Resistivity logs (LLD/LLS), processed using the Techlog software platform. This evaluation aimed to characterize key reservoir properties, including volume of shale (Vsh), effective porosity (Φeff), water saturation (Sw), and net pay thickness, providing essential insights into the hydrocarbon potential and quality of the AEB-3E reservoir.

Shale volume was estimated from the Gamma Ray log using the linear method. The calculated Vsh values across the AEB-3E sub-member revealed generally low shale content, with values ranging from approximately 3–8%. Notably, Vsh slightly increases toward the central part of the study area, reaching up to 8% in the Berenice-TD-1X well. This trend suggests mild shale enrichment in deeper basin areas, which remains within acceptable limits for good reservoir quality. Conversely, shale volumes decrease toward the SE and SW directions, reaching minimum values of around 3–4%, indicating cleaner sandstone facies in those regions.

Effective porosity was calculated by correcting the total porosity (derived from Density-Neutron logs) for the effect of shale content. The results showed a clear spatial trend across the field. In the AEB-3E sub-member, Φeff reaches up to 18% in the southeastern part of the field, reflecting highly porous intervals conducive to hydrocarbon storage and flow. Porosity gradually decreases towards the northwestern direction, with values around 10%, suggesting potential degradation in reservoir quality. These lateral variations are likely influenced by facies heterogeneity and depositional energy gradients across the field.

Water saturation was determined using the Indonesian model due to the presence of dispersed shale. The calculated Sw values showed a notable spatial pattern, with water saturation increasing toward the western part of the area, reaching 54%. In contrast, lower saturation values were observed in the SE region (around 28%), where effective porosity is highest. This inverse relationship between porosity and Sw is consistent with typical reservoir behavior, and it reinforces the interpretation that the SE sector of the Berenice field holds the most promising hydrocarbon-bearing zones.

**Table 1:** Detailed evaluation of petrophysical properties across the study area.

Well	Zones	Unit	Gross	Net	Vsh Fraction	PHIE Fraction	Sw Fraction
Berenice – TD-1X	AEB-3E	FT	549	203	0.078	0.133	0.288
BERENICE-03	AEB-3E	FT	221	44	0.07	0.117	0.328
BERENICE-08	AEB-3E	FT	146	100	0.047	0.142	0.450
BERENICE-09	AEB-3E	FT	176	75	0.056	0.147	0.374

### Net-pay calculation

Estimating net pay is a critical aspect of reservoir evaluation, as it defines the intervals capable of producing hydrocarbons economically^11,16,18,30,31^. In the Berenice Oil Field, this process involved integrating petrophysical parameters with hydrocarbon detection indicators to isolate the reservoir portions with optimal quality and fluid content. Net-pay intervals were identified by applying appropriate cut-off values to parameters such as effective porosity, water saturation, and shale volume. These thresholds were selected based on detailed petrophysical evaluations and guided by indicators like neutron-density crossover, deep versus shallow resistivity separation, and movable hydrocarbon plots. This multi-parameter filtering ensures that only reservoir sections with adequate porosity, low shale content, and sufficient hydrocarbon saturation are considered productive.

This approach ensures a consistent alignment between net-pay estimation and previously interpreted hydrocarbon-bearing zones. Net-pay mapping across the wells highlights how reservoir productivity varies laterally and vertically, which is vital for resource quantification, development planning, and well placement optimization. By isolating these high-potential intervals, a more accurate assessment of the reservoir’s economic viability is achieved.

**Fig. 11:**
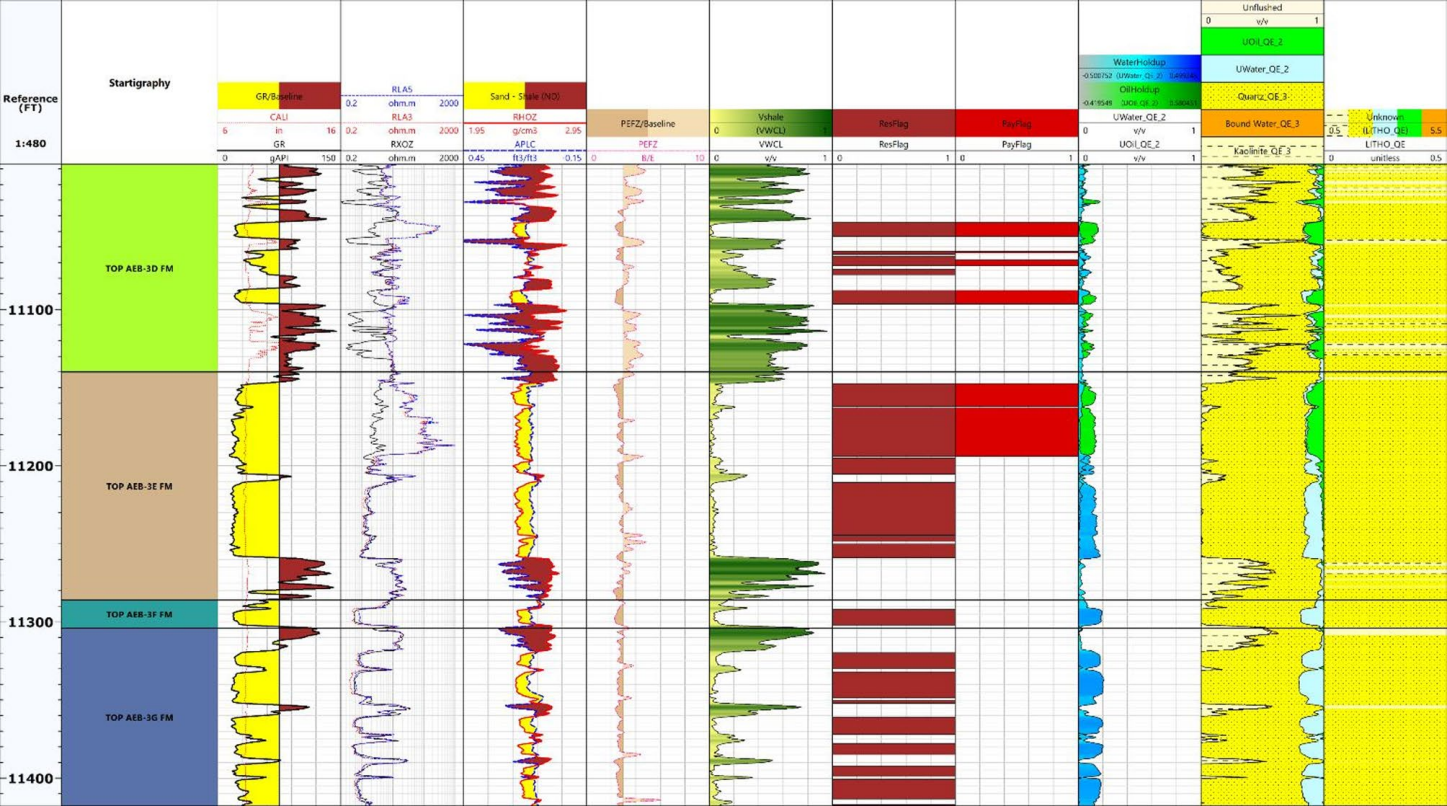
Detailed Estimation and Delineation of Net Pay Zones within the Berenice-08 Well Based on Integrated Petrophysical Analysis.

The calculated net-pay zone closely corresponds to the hydrocarbon-bearing interval previously identified between depths of 11,150 and 11,190 ft. This alignment reinforces the reliability of the hydrocarbon detection methods used, namely the neutron-density crossover, resistivity separation, and movable hydrocarbon evaluation. The application of precise cut-off criteria helped delineate the productive zone with high confidence, confirming that this interval represents a reservoir section with both suitable rock properties and hydrocarbon presence. This congruence between hydrocarbon indicators and net-pay estimation further validates petrophysical interpretation and provides a robust foundation for future development decisions in the Berenice Oil Field (Figure. 11).

### Reservoir heterogeneity and continuity

**Fig. 12:**
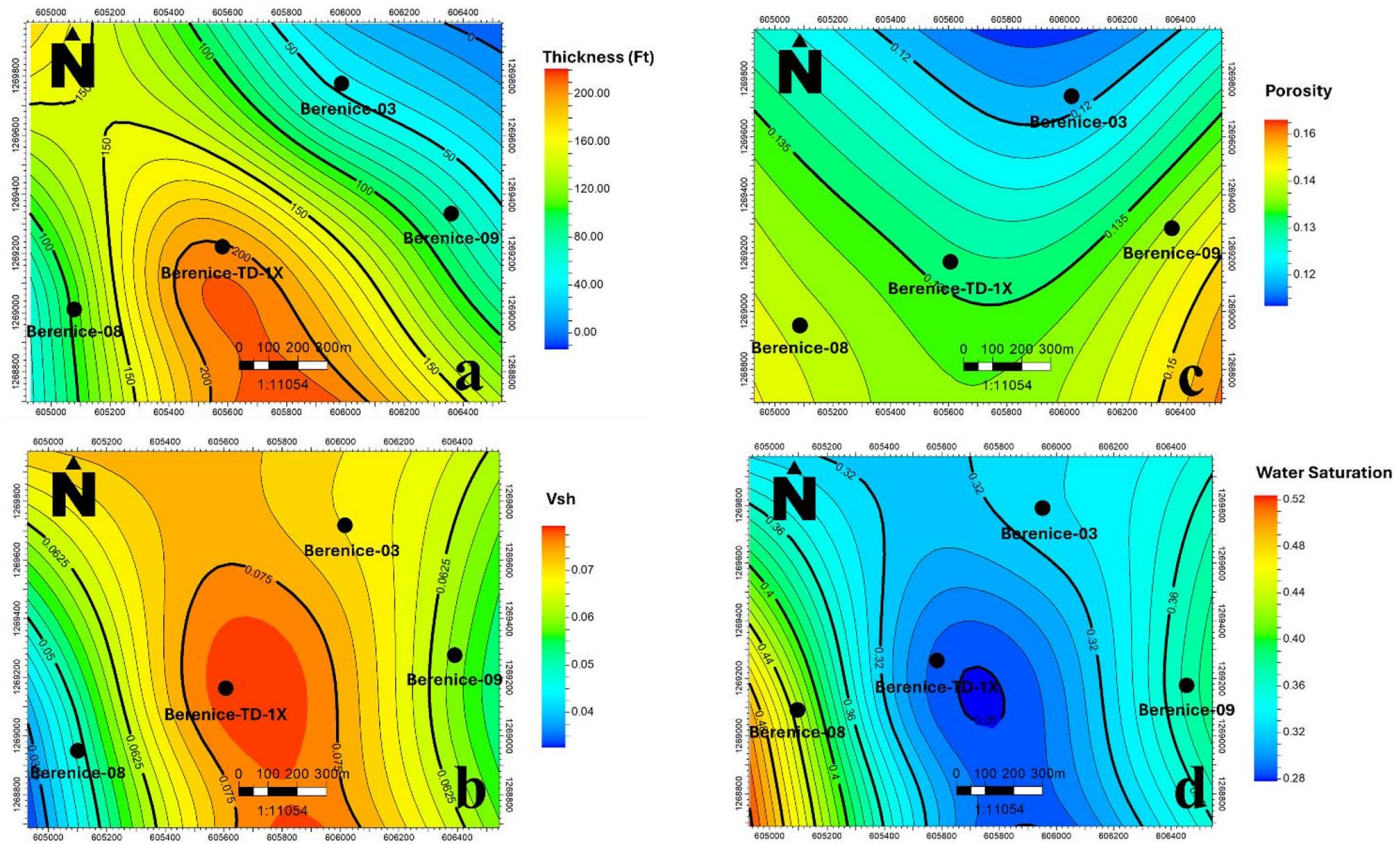
Spatial distribution maps illustrate key petrophysical properties of the AEB-3E sub-member across the four wells in the Berenice Oil Field: (**a**) Formation thickness, (**b**) Volume of shale, (**c**) Effective porosity, and (d) Water saturation.

To assess the lateral variability of reservoir quality, spatial distribution maps were created using the Inverse Distance Weighting (IDW) interpolation technique, chosen for its practicality and effectiveness given the limited dataset for key petrophysical properties of the AEB-3E sub-member across the four wells in the Berenice Oil Field (Figure. 12). These maps display formation thickness, shale volume, effective porosity, and water saturation, offering a comprehensive view of reservoir heterogeneity and helping to pinpoint areas with favorable reservoir characteristics. By visualizing these properties across the study area, the maps serve as essential tools for understanding changes in reservoir behavior, quality, and fluid distribution. They provide a clear representation of how depositional and diagenetic processes have impacted the reservoir laterally, supporting better planning for field development, drilling strategies, and reservoir management.

Analysis of the petrophysical maps revealed that the AEB-3E sub-member exhibits its maximum thickness of approximately 170 ft in the central part of the field at the Berenice-TD-1X well. This thickness gradually reduces toward the flanks, reaching a minimum of about 40 ft. Correspondingly, shale volume shows a gentle increase in the central area, reaching around 8%—a relatively low value—while it decreases toward the southeast and southwest, where values drop to approximately 3–4%.

Effective porosity exhibits a notable trend, increasing toward the southern part of the field, particularly in the southeast direction, where it peaks at around 18%. In contrast, it declines to nearly 10% in the northern sector. Water saturation mapping indicates that saturation levels rise toward the western part of the area, reaching about 54%, while the lowest values, around 28%, are observed in the southeast.

This correlation between low water saturation and high effective porosity in the southeastern direction highlights it as a highly prospective zone. These findings suggest that the southeast portion of the Berenice Field offers the most favorable conditions for future well placement, with improved reservoir quality and fluid saturation levels aligned to support enhanced hydrocarbon recovery.

**Fig. 13:**
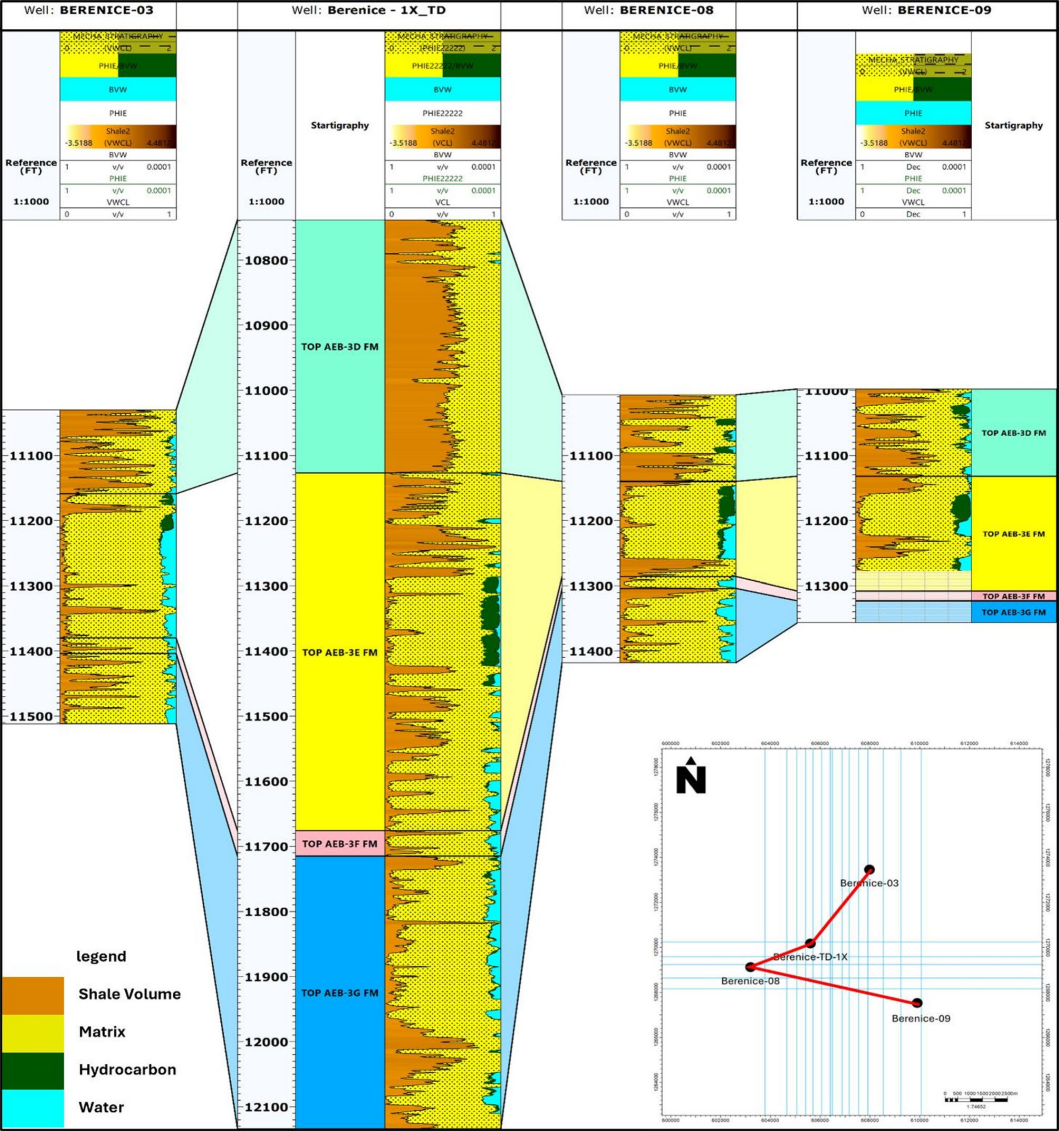
Integrated rock model displaying corrected Volume of Shale, Effective Porosity, Bulk Volume of Water, and Bulk Volume of Hydrocarbons in a single track; alongside well correlation across the Berenice Oil Field to evaluate the lateral continuity and variability of the AEB-3E sub-member and related interval.

A comprehensive rock model was constructed by displaying the corrected Volume of Shale alongside Effective Porosity, Bulk Volume of Water, and Bulk Volume of Hydrocarbons within a single track. This integrated visualization is highly valuable as it highlights the relative proportions of mineral matrix and fluids throughout the reservoir intervals along each well. Such a display aids in understanding the heterogeneity of formation constituents and fluid content, allowing for more precise reservoir characterization and facilitating better decision-making for exploration and development. Figure. 13.

Correlation of wells throughout the Berenice Oil Field was conducted to assess the lateral persistence and variability of the AEB-3E sub-member and its associated intervals (Figure. 13). By aligning formation tops and comparing key petrophysical attributes across multiple wells, it became possible to track changes in thickness and distribution patterns of the reservoir units. This process aids in interpreting depositional trends and understanding structural influences on reservoir geometry, both of which are vital for guiding field development and well planning^43,44^.

The correlation revealed that the AEB-3E sub-member displays relatively uniform thickness across most wells. However, a noticeable thickening occurs in the Berenice-TD-1X well, located near the center of the study area. This observation suggests that the basin deepens toward the central part, resulting in a thicker sedimentary package and potentially more favorable conditions for reservoir development in that region.

### Economic implications of the southeastern High-Porosity zone

The southeastern part of the Berenice Field, particularly within the AEB-3E sub-member, exhibits the most favorable reservoir characteristics, making it a strong candidate for economically viable hydrocarbon production. Petrophysical analysis reveals that this zone has effective porosity values reaching up to 18%, low shale volume (as low as 3–4%), and minimum water saturation values around 28%, indicating excellent reservoir quality and high hydrocarbon saturation.

The net pay thickness in this zone is also considerable, as observed in wells such as Berenice-08 and Berenice-TD-1X, where net pay reaches 100 ft and 203 ft, respectively. These characteristics are indicative of zones capable of sustaining commercial hydrocarbon flow rates with minimal formation damage or water production risk.

From an economic standpoint, such high-quality intervals reduce the need for extensive stimulation or artificial lift, thus lowering development costs. Moreover, the spatial continuity of these high-porosity zones—confirmed by well correlation and seismic interpretation—enhances the potential for cluster well development, improving field development efficiency.

In terms of recoverable reserves, the combination of thick net pay, high effective porosity, and favorable saturation profiles suggests that the southeastern sector could contain a significant proportion of the field’s total hydrocarbon volumes. When tied with existing infrastructure in the Berenice Field, this area becomes highly attractive for near-term production planning and incremental reserve additions.

Additionally, this zone holds promise for dual-purpose development: it not only presents a viable target for continued hydrocarbon extraction but also exhibits strong geological sealing and petrophysical containment properties, making it suitable for future CO₂ storage projects, further enhancing its long-term economic and environmental value.

### Uncertainty in Log-Derived parameters

Despite the robust results obtained from the petrophysical evaluation, it is important to acknowledge the inherent uncertainties associated with log-derived parameters, particularly water saturation (Sw). The calculation of Sw relied on the Archie equation and the Indonesian model, both of which involve empirical assumptions. Archie’s method, applied in zones interpreted as clean sandstone, assumes uniform lithology, constant cementation and saturation exponents, and the absence of conductive minerals conditions that may not be fully met in the AEB-3E reservoir. Although the formation is predominantly composed of sandstone, the presence of interbedded shale and siltstone, as indicated by RHOB–NPHI and M–N crossplots, introduces variability that may impact resistivity readings. In intervals with increased clay content, the Indonesian model was employed to better account for shale conductivity; however, variations in clay type and distribution can still influence the accuracy of Sw estimates. Additionally, the Archie parameters (a = 1, m = 1.9, *n* = 2) were derived from Pickett plot analysis using data from a single well (Berenice-08), and their application across the entire field assumes lateral homogeneity in formation properties. While these approaches provide reasonable approximations in the absence of core measurements, the potential deviations should be considered when interpreting saturation and volumetric estimates. Recognizing these limitations enhances the reliability of the study’s conclusions and emphasizes the importance of integrating multiple data sources where possible in future work.

### Seismic interpretation

**Fig. 14:**
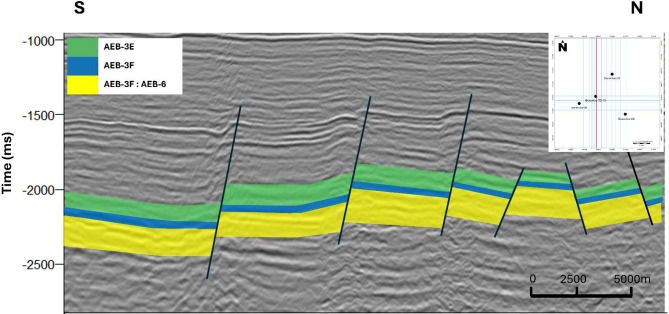
Seismically interpreted section of Crossline 5, highlighted on the accompanying base map, illustrating structural styles within the Berenice Field along with the legend of interpreted horizons^18^.

Seismic interpretation of the Berenice Oil Field by using Petrel software-2017 reveals that the dominant trap mechanism in the area is structural trapping^18,23,45–47^. The subsurface is characterized by a series of normal faults trending ENE–WSW^48–50^which segment the reservoir into a pattern of step faults, forming alternating horst and graben blocks. These faulted structures create favorable conditions for hydrocarbon entrapment, with the horsts often acting as structural highs that serve as potential hydrocarbon-bearing compartments. The continuity and orientation of these fault systems are crucial in defining the extent and boundaries of the reservoir, as they provide the necessary closure for hydrocarbon accumulation, particularly in the targeted AEB-3E sub-member. Figure 14 shows the interpretation of seismic section within Berenice Field.

### Petroleum system elements

The petroleum system in the Berenice area is well-developed and supports active hydrocarbon generation and entrapment. The AEB-3E sub-member serves as the primary reservoir rock, characterized by good porosity and permeability. The Safa Formation is considered a key source rock within the Western Desert petroleum system, particularly in the Faghur Basin and adjacent areas. Previous geochemical studies have indicated that the formation contains intervals with fair to good hydrocarbon generation potential. Total Organic Carbon (TOC) values range from 1.2 to 3.5%, while vitrinite reflectance (Ro) values between 0.7% and 1.1% suggest thermal maturity levels corresponding to the early to peak oil generation window^51,52^. These findings are consistent with the regional geological framework and exploration data confirming the Safa Formation’s role as an effective source rock within the established petroleum system of the Western Desert. The seal rocks are predominantly intraformational shales within the AEB sequence, while regional evaporites and tight shales from overlying formations act as effective cap rocks, preventing vertical migration. This combination of source, reservoir, and seal elements ensures a functioning and efficient petroleum system within the Berenice Oil Field.

## Conclusion

This study presents a comprehensive petrophysical evaluation of the Alam El Bueib 3E (AEB-3E) reservoir within the Berenice Oil Field, emphasizing its reservoir quality, hydrocarbon potential, and spatial continuity to support optimized field development. The AEB-3E Member is identified as a principal sandstone reservoir exhibiting variable petrophysical characteristics influenced by facies heterogeneity and structural complexity.

Using wireline log data from four wells and advanced petrophysical workflows, key reservoir properties including lithology, porosity, water saturation, and net pay were systematically characterized. Crossplot analyses (M-N and RHOB-NPHI) confirmed that the AEB-3E reservoir is predominantly composed of clean sandstone interbedded with minor siltstone and shale, enabling accurate assessment of reservoir quality and fluid saturations. Hydrocarbon presence was validated by integrating neutron-density crossover, resistivity separation, and movable oil plot methods, which delineated productive intervals primarily between depths of 11,150 and 11,190 ft. In the absence of core data, the Pickett plot analysis on Berenice-08 well provided reliable estimates of formation water resistivity (Rw = 0.0378) and Archie parameters (a = 1, m = 1.9, *n* = 2), which were critical for precise water saturation calculations.

Petrophysical results indicate low shale volume (3–8%) and effective porosity values reaching up to 18%, particularly in the southeastern sector, which also corresponds to the lowest water saturation (28%), marking this area as the most prospective hydrocarbon-bearing zone within the field. Net pay was rigorously quantified through multi-parameter cut-offs aligned with hydrocarbon indicators, confirming reservoir intervals consistent with fluid presence and quality, thereby providing a robust basis for future development decisions. Spatial distribution maps further illustrated lateral reservoir heterogeneity, showing maximum formation thickness and slightly increased shale content centrally, while highlighting the southeastern region for enhanced porosity and hydrocarbon saturation.

A rock model integrating shale volume, effective porosity, and fluid volumes was developed, offering a detailed visualization of reservoir heterogeneity and supporting more informed exploration and development strategies. A well correlation across the field demonstrated generally uniform thickness of the AEB-3E unit, with notable thickening at the central Berenice-TD-1X well, indicating structural control on sediment accumulation and reservoir potential.

Seismic interpretation revealed that structural trapping, governed by ENE–WSW trending normal faults forming horst and graben blocks, is the dominant hydrocarbon trap mechanism. The petroleum system is well-developed, comprising mature source rocks from the Upper and Lower Safa formations, intraformational shale seals, and regional cap rocks, collectively ensuring effective hydrocarbon generation, migration, and entrapment.

Overall, this integrated petrophysical and structural analysis provides critical insights into Berenice Field’s reservoir architecture and fluid distribution, enabling targeted reservoir management and optimized hydrocarbon recovery.

## Data Availability

The data supporting the findings of this study are provided by the Egyptian General Petroleum Corporation. However, due to licensing restrictions, these data are not publicly accessible. They can be obtained from the corresponding author upon reasonable request and with prior approval from the Egyptian General Petroleum Corporation.
